# Supplementary material for: Effect of Inhalation Profile on Delivery of Treprostinil Palmitil Inhalation Powder
Source: Pharmaceutics. 2023 Mar 14;15(3):934. doi: 10.3390/pharmaceutics15030934 (PMC10057251; doi:10.3390/pharmaceutics15030934)
Supplement: Supplementary file 1 [file pharmaceutics-15-00934-s001.zip › pharmaceutics-2223479-supplementary.pdf]

Supplemental Figures and Tables:

Figure S1. The square root of the pressure drop is plotted as a function of flow rate for the High Resistance device [10].

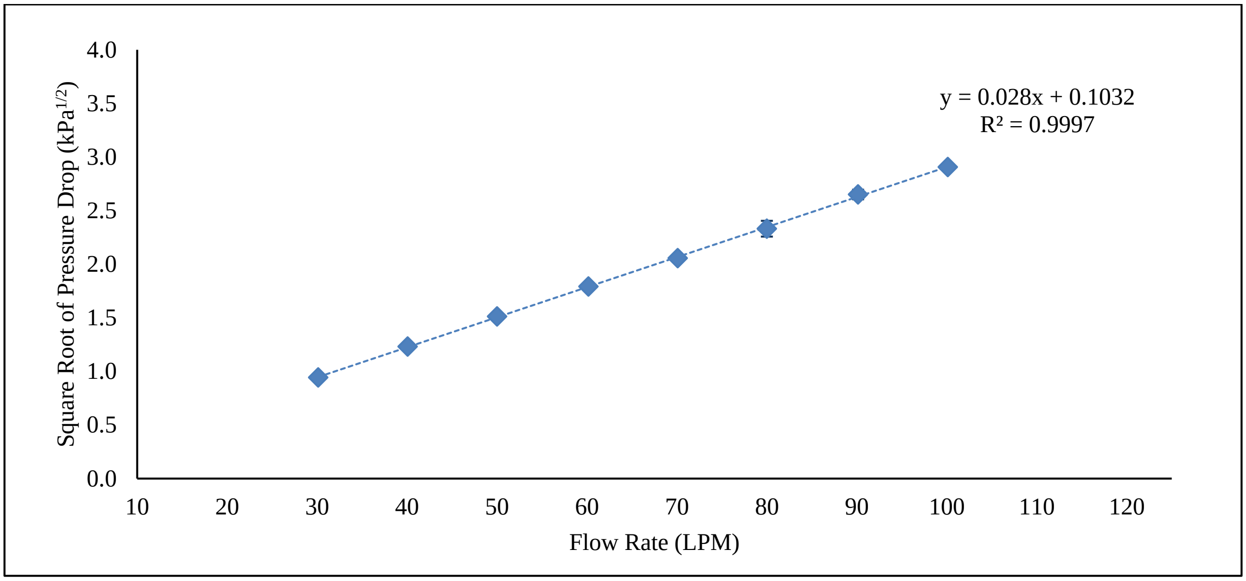

Figure S2. Breath Simulator and NGI Setup.

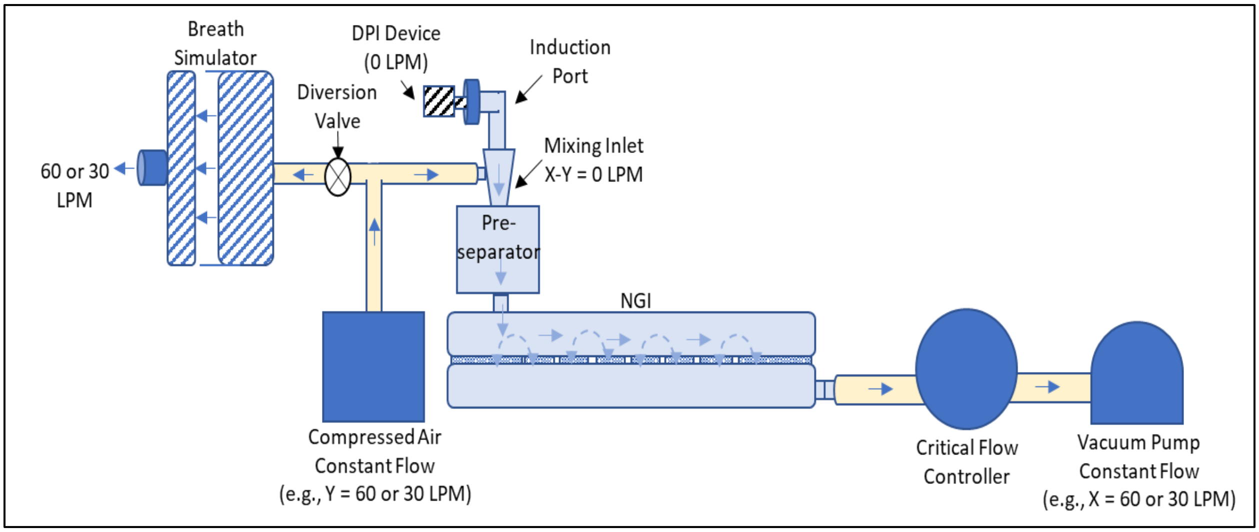

Table S1. BRS system trapezoidal waveform test scripts [ramp time, inhalation volume] to reach 60 LPM (1030 mL/s) and 30 LPM (510 mL/s) peak inspiratory flow rates.

50 ms, 1 L		50 ms, 2 L		50 ms, 4 L	
Time (s)	Flow Rate (mL/s)	Time (s)	Flow Rate (mL/s)	Time (s)	Flow Rate (mL/s)
0.000	0	0.000	0	0.000	0
0.050	1030	0.050	1030	0.050	1030
0.996	1030	1.967	1030	3.909	1030
1.096	0	2.067	0	4.009	0
170 ms, 4 L		500 ms, 4 L		1000 ms, 4 L	
Time (s)	Flow Rate (mL/s)	Time (s)	Flow Rate (mL/s)	Time (s)	Flow Rate (mL/s)
0.000	0	0.000	0	0.000	0
0.170	1030	0.500	1030	1.000	1030
3.970	1030	4.134	1030	4.384	1030
4.070	0	4.234	0	4.484	0
2000 ms, 1 L		2000 ms, 2 L		2000 ms, 4 L	
Time (s)	Flow Rate (mL/s)	Time (s)	Flow Rate (mL/s)	Time (s)	Flow Rate (mL/s)
0.000	0	0.000	0	0.000	0
1.942	1030	2.000	1030	2.000	1030
2.042	0	2.942	1030	4.884	1030
-	-	3.042	0	4.984	0
25 ms, 1 L		25 ms, 2 L		25 ms, 4 L	
Time (s)	Flow Rate (mL/s)	Time (s)	Flow Rate (mL/s)	Time (s)	Flow Rate (mL/s)
0.000	0	0.000	0	0.000	0
0.025	510	0.025	510	0.025	510
1.976	510	3.935	510	7.857	510
2.076	0	4.035	0	7.957	0
1000 ms, 1 L		1000 ms, 2 L		1000 ms, 4 L	
Time (s)	Flow Rate (mL/s)	Time (s)	Flow Rate (mL/s)	Time (s)	Flow Rate (mL/s)
0.000	0	0.000	0	0.000	0
1	510	1	510	1	1030
2.464	510	4.423	510	8.344	1030
2.564	0	4.523	0	8.454	0

Table S2. Additional aerosol performance characteristics (i.e., eFPF, eFPD and total recovery) for the 16 and 32 mg TPIP capsules as a function of inhalation flow rate ramp times: 60 LPM peak inspiratory flow rate and 4 L inhalation volume. eFPF= Extra Fine Particle Fraction; eFPD=Extra Fine Particle Dose; TP= Treprostinil Palmitil

Inhalation Ramp Time	n	eFPF (< 2 µm)		eFPD (< 2 µm)	Total Recovery	
		% of Emitted Dose	% of Loaded Dose	µg, TP	%	µg, TP
16 mg capsule containing 160 µg TP						
50 ms	3	58.0 ± 1.9	46.3 ± 2.1	74.11 ± 3.41	90.9 ± 0.6	145.42 ± 1.01
170 ms	3	58.8 ± 1.0	47.8 ± 0.4	76.53 ± 0.69	93.1 ± 0.8	148.99 ± 1.26
500 ms	3	62.5 ± 0.2	49.9 ± 0.6	79.79 ± 1.01	92.4 ± 1.2	147.88 ± 1.88
1000 ms	3	62.7 ± 0.9	50.4 ± 2.3	80.60 ± 3.71	92.7 ± 1.6	148.33 ± 2.51
2000 ms	3	62.0 ± 1.3	50.7 ± 1.5	81.16 ± 2.33	94.9 ± 0.9	151.77 ± 1.39
32 mg capsule containing 320 µg TP						
50 ms	3	48.0 ± 1.3	39.0 ± 1.1	124.96 ± 3.59	90.0 ± 1.6	288.06 ± 5.23
170 ms	3	49.8 ± 2.3	41.2 ± 2.4	131.88 ± 7.76	92.5 ± 2.0	296.11 ± 6.40
500 ms	3	52.7 ± 1.5	42.8 ± 1.0	136.97 ± 3.35	90.9 ± 1.0	290.81 ± 3.13
1000 ms	3	52.9 ± 2.6	43.9 ± 2.3	140.42 ± 7.50	92.6 ± 0.4	296.15 ± 1.38
2000 ms	3	54.4 ± 2.2	48.7 ± 3.1	155.83 ± 9.96	98.7 ± 1.9	315.89 ± 6.16

Table S3. Additional aerosol performance characteristics (i.e., eFPF, eFPD and total recovery) for the 16 and 32 mg TPIP capsules as a function of inhalation volume (1, 2 and 4 L) and acceleration rate (50 and 2000 ms flow rate ramps for the 60 LPM, and 25 and 1000 ms flow rate ramps for the 30 LPM peak inspiratory flow rates). eFPF= Extra Fine Particle Fraction; eFPD=Extra Fine Particle Dose; TP= Treprostinil Palmitil

Inhalation Parameters: ramp time, inhaled volume	n	eFPF (< 2 µm)		eFPD (< 2 µm)	Total Recovery	
		% of Emitted Dose	% of Loaded Dose	µg, TP	%	µg, TP
16 mg capsule containing 160 µg TP at 60 LPM (50 and 2000 ramp times corresponding to 20 and 0.5 L/s^2 acceleration rates, respectively)						
50 ms, 1 L	3	57.1 ± 0.9	46.3 ± 0.4	74.09 ± 0.70	93.8 ± 1.0	150.08 ± 1.66
50 ms, 2 L	3	57.2 ± 0.6	45.2 ± 0.6	72.40 ± 0.95	92.0 ± 1.2	147.18 ± 1.88
*50 ms, 4 L	3	58.0 ± 1.9	46.3 ± 2.1	74.11 ± 3.41	90.9 ± 0.6	145.42 ± 1.01
2000 ms, 1 L	3	58.5 ± 1.2	48.2 ± 1.1	77.05 ± 1.82	96.5 ± 1.4	154.42 ± 2.31
2000 ms, 2 L	3	61.2 ± 1.0	50.5 ± 2.2	80.74 ± 3.49	95.6 ± 1.9	152.97 ± 3.11
*2000 ms, 4 L	3	62.0 ± 1.3	50.7 ± 1.5	81.16 ± 2.33	94.9 ± 0.9	151.77 ± 1.39
32 mg capsule containing 320 µg TP at 60 LPM (50 and 2000 ramp times corresponding to 20 and 0.5 L/s^2 acceleration rates, respectively)						
50 ms, 1 L	3	44.9 ± 0.8	37.7 ± 0.6	120.78 ± 1.99	94.8 ± 1.0	303.26 ± 3.28
50 ms, 2 L	3	46.7 ± 1.0	39.5 ± 0.7	126.52 ± 2.16	94.8 ± 1.2	303.31 ± 3.97
*50 ms, 4 L	3	48.0 ± 1.3	39.0 ± 1.1	124.96 ± 3.59	90.0 ± 1.6	288.06 ± 5.23
50 ms, 4 L	3	45.8 ± 1.4	38.4 ± 1.2	122.82 ± 3.93	92.7 ± 0.2	296.56 ± 0.76
2000 ms, 1 L	3	54.4 ± 1.6	44.2 ± 0.7	141.29 ± 2.13	93.7 ± 1.5	299.82 ± 4.88
2000 ms, 2 L	3	54.0 ± 1.0	46.8 ± 0.7	149.90 ± 2.23	95.0 ± 0.6	303.95 ± 2.02
*2000 ms, 4 L	3	54.4 ± 2.2	48.7 ± 3.1	155.83 ± 9.96	98.7 ± 1.9	315.89 ± 6.16
2000 ms, 4 L	3	53.7 ± 0.3	47.0 ± 0.7	150.43 ± 2.11	96.5 ± 0.4	308.87 ± 1.32

* Original data from the acceleration rate study for the 16 mg and 32 mg capsule. No significant differences ($p > 0.05$) in the ED, MMAD, FPF, and FPD between the original and repeat runs.
